# *"They don't care what happens to us." *The situation of double orphans heading households in Rakai District, Uganda

**DOI:** 10.1186/1471-2458-9-321

**Published:** 2009-09-04

**Authors:** Nina Dalen, Ann Jacqueline Nakitende, Seggane Musisi

**Affiliations:** 1 Department of Radiography, Faculty of Health and Social Sciences, Bergen University College and Centre for International Health, University of Bergen, Norway; 2 Department of Psychiatry, Makerere University, Kampala, Uganda; 3 Department of Psychiatry, Makerere University School of Medicine and Mulago Hospital, Kampala, Uganda

## Abstract

**Background:**

This article is based on information collected about the situation of double orphans who are heading households in Rakai District, Uganda. The information will be used as justification and guidance for planning actions to improve the situation of these and similar children. This research is thus the first step in an Action Research approach leading to specific interventions. The aim of this article is to describe the situation of these orphaned children, with an emphasis on the psychosocial challenges they face.

**Methods:**

The study involved interviews, focus group discussions, observations and narratives. Forty-three heads of sibling-headed households participated. Information derived from informal discussions with local leaders is also included. The responses were analyzed using a modified version of Giorgi's psychological phenomenological method as described by Malterud [1].

**Results:**

Factors such as lack of material resources, including food and clothes, limited possibilities to attend school on a regular basis, vast responsibilities and reduced possibilities for social interaction all contribute to causing worries and challenges for the child heads of households. Most of the children claimed that they were stigmatized and, to a great extent, ignored and excluded from their community. The Local Council Secretary ("Chairman") seemed to be the person in the community most responsible and helpful, but some chairmen seemed not to care at all. The children requested counseling for themselves as well as for community members because they experienced lack of understanding from other children and from adult community members.

**Conclusion:**

The children experienced their situation as a huge and complex problem for themselves as well as for people in their villages. However, the situation might improve if actions focused on practical and psychological issues as well as on sensitization about the children's situation could be initiated. In addition to the fact that these children need adult guidance to become citizens who act in accordance with the expectations in their communities, material aid is important in order to reduce the children's experiences of being "different" and constantly experiencing survival anxieties.

Before my parents died, I was schooling without facing any problems and my heart was at rest. When they died I went to live with Jjajja [grandmother]. She fell very sick and I came out of school for a full term to look after her. I was treating Jjajja but she was not getting better. She died...so...I got my schoolmates' books and copied notes that they had taken while I was away from school...I face the problem of not having good friends. Some see me as a disease...other people are not bad. Some call me names and say that I am stupid, that I probably inherited the stupidity from my mother or father...Ever since my parents died, I have not had peace. I spend most of the time thinking, crying and struggling within myself asking God why He really had to do such a thing and saying to myself that: "God, help me overcome these problems!"

*Girl, 15*.

## Background

In this article, we attempt to describe the situation of double orphans (having lost both parents) who are heading sibling-headed households in Rakai District, Uganda. The term "sibling-headed households" is used because we have included young adult orphans who were under the age of 18 when they started acting as breadwinners after both parents had died. Their social, physical and material needs were investigated and we aimed to emphasize the psychosocial challenges that these unmet needs might have caused, as well as the psychological impact caused by the deaths of both parents. It was also important to investigate to what extent orphans in sibling-headed households experienced "belonging" in their community.

In this article, "psycho" refers to emotions, behavior, thoughts and attitudes of the child and "social" refers to the child's external relationships and to the influences of the social environment and social change on the child's daily life, see page 3 [2].

Approximately 2 million (or 1 in 5) children are orphaned in Uganda, about half of them because of HIV/AIDS [3,4]. Rakai District is located in the South Western region of Uganda and has one of the highest rates of HIV/AIDS in the country. Between 35 000 and 50 000 out of a total number of 267 000 children in Rakai District are orphaned due to AIDS [5,6]. Sibling-headed households were noted in Rakai District already in the late 1980s [7,8]. According to the Rakai District Local Government [6] there were 969 sibling-headed households in the district in 2003, each having an average of 3.3 orphans, and the number is increasing [6,8-10].

The HIV/AIDS prevalence in Uganda peaked in 1991 with 15% among adults and over 30% among pregnant women. The national prevalence was brought down to around 6% in 2001 due to an intensive campaign initiated by the government [11]. However, the prevalence has increased from 6.1% in 2004 to 6.4% in 2006 and continues to increase slowly [3]. This means that as the total population in Uganda increases, an increasing number of people will be infected and affected. Communities with high rates of HIV infection have for many years experienced a rapid increase in the number of children becoming orphaned [12]. Because exposure to HIV/AIDS is a prevalent hazard in countries such as Uganda, and because of the relatively poor capacity, methods and possibility of treatment in low income countries [13], the probability is high that a single orphan (a child who has lost one parent) will also lose the other parent. Therefore regions and countries with high levels of HIV/AIDS will continue to have an increasingly higher number of double orphans as the pandemic advances.

More than 33% of the children in Rakai District are orphans - significantly higher than the country's average. Slightly below one percent of all the 267 000 children are double orphans. One percent of all the 92, 160 households are child-headed and 75% of them are headed by a male orphan. Orphans constitute about half of all vulnerable children. Vulnerable children are those with a "high probability of a negative outcome, or an expected welfare loss above a socially accepted norm", see page 13 [14]. At the national level, 45.3% of all children are vulnerable; the percentage of such children in Rakai District is 48.8 [15]. Perhaps these numbers of double orphans heading households do not seem high, but these children and the siblings whom they care for are the most vulnerable of the vulnerable [14,16] and therefore this study has focused on them. "Orphans not accommodated within the family of adults have to look after themselves, their siblings, and, in some cases, their elder relatives. Child-headed households are likely to have poor housing, [poor] sanitation hygiene and malnutrition", see page 119 [17].

According to the Rakai District Local Government, one of the reasons for the increasing number of orphans and other vulnerable children living on their own is the combination of poverty and the increasing intra-household dependency ratios that has eroded socio-cultural values [15] such as the extent to which relatives take care of orphans. A second reason many double orphans keep living in their past parents' home is that these children are afraid that other people/relatives will take their property--as has occurred with some frequency [4]. Still others stay at home because they are not welcome in any other places and also fear they will become house servants, and treated differently than other children in the same household. [7,10].

Formerly in Uganda, community members and extended families absorbed the burden of orphans. The prevailing socio-cultural values made it natural that orphaned children should primarily be cared for within their extended families; community settings such as institutionalized care was presumed not to be good for children and to increase stigma of the child [18]. In addition, the care in many of these orphanages tends to be of poor quality for the emotional life of the children [18,19]. Presently, the extended families remain the principle orphan-care units but due to the greatly increased number of orphans, in some regions the families' abilities to care for orphans seem to have reached the maximum elasticity of absorption which, in turn, has led to the growth of sibling headed households [6,8].

The majority of studies of orphans in sub-Saharan Africa have focused on physical and socioeconomic factors, such as access to education, food, shelter and clothing, factors which are observable and therefore easier to address. As a result, psychosocial needs have received less attention. Nonetheless, research dedicated to describing these orphans indicate that studies of their psychosocial and developmental needs is warranted [4,9,20,21], especially in resource-poor countries [22]. To date, only a few studies have examined the psychosocial dimensions of orphanhood in Uganda. The exceptional situation of the young people who have to take over as the breadwinners of their families after their parents' death long before they are physically, mentally and emotionally prepared to do so, is hardly discussed.

Sengendo and Nambi [23] carried out a study on the psychological effects of orphanhood among orphans in Rakai District. Since their study was mainly funded by the international Non-Governmental Organization (NGO) World Vision (WV), their study population consisted of orphans receiving education sponsorship from WV, which meant that most of the children were attending school. They found that depressive thoughts and feelings such as sadness, anger and guilt were present in the children at the time of bereavement. They also argued that as the individual child, over time, accepts the loss, "the negative emotions are expected to disappear", see page 115 [23]. Atwine et al. [9] have compared psychological distress among orphans and non-orphans in the rural Bushenyi District in Uganda. According to their findings, orphans who had lost one or both parents ran greater risks than non-orphans of having higher levels of anxiety, depression and anger. However, this result may have been influenced by rather leading questions, such as "Do you think that your life will be bad?" They also argue that depression scores were higher in orphans living in smaller vs. larger households. Musisi, Kinyanda & Nakigudde [24] argue that orphans in Rakai District reported more dissatisfaction with life, and were more emotionally needy and isolated, than non-orphans. Their study, however, focused on children in school only and does not report whether the orphans lived on their own or in other families.

In this article, we describe the situation of double orphans who are heading sibling-headed households. Our specific objectives are: 1) To describe the sibling-headed households' composition in relation to number of siblings, age, food and maintenance, health care and cause of parents' death; 2) to document the heads of households' school attendance, work load and responsibilities; 3) to indicate the heads of households' social interaction with other children, relatives and community members; 4) to describe the psychosocial challenges that these issues might have caused; 5) to reveal the heads of households' experience of "belonging" in their community and 6) to suggest possible ways these challenges can be met.

A great amount of the literature on orphans point to the fact that comparatively, and in general, the situation of double orphans living in sibling-headed households is among the worst created by the AIDS epidemic [14,16,25,26]. For this reason, we attempted to understand the situation in sibling headed households in order to initiate interventions for improvement.

## Methods

The data for this article was collected in eight different villages in three counties - Kooki, Kyotera and Kakuuto - in Rakai District. This district borders Lake Victoria and western Tanzania in the South. Approximately 95% of the 471.806 inhabitants live in rural areas, and 70% of the households survive on less than UgShs 5000 (= US$ 3) per week [6,10,27]. Rakai District's HIV prevalence rate of 12 -15% is twice as high as the national rate of 6 - 7% [6,28]. Ethnically the district is diverse, with the Baganda as the dominant ethnic group. Other ethnic groups include Banyankole, Barundi, Baziba and Banyambo, and also a large number of people of Rwandanese origin due to the Rwandan genocide in 1994 [6].

Uganda has a five-tiered local administrative system of elected Local Councils (LCs) and executive committees. LC1 is responsible for the villages, and the other LCs are respectively responsible for the parishes, the sub-counties, the municipalities and LC5 for the districts. Each local council at every level includes an executive committee of nine members. The members of LC1 and LC2 are elected volunteers and not paid. The term "chairman" in this article refers to the secretary (leader) of the LC1 committee in a village. In local parlance, he is commonly referred to as either simply "The LC" or as "Chairman".

Because knowledge produced in research should be beneficial to the participants, and harmful consequences of research eliminated or minimized [29], we carefully informed all participants by presenting the research and the informed consent forms both orally and written in English and in Luganda. Signed or thumb printed informed consent was obtained from all respondents. The Uganda National Council for Science and Technology (UNCST) approved the study. Permission and letters of introduction from the UNCST were presented to the District Director of Health Services in Rakai District and to local leaders.

In order to describe the variety of challenges faced by child heads of households, a broad methodological approach was used. The study employed qualitative methods because we wanted to obtain the children's own descriptions of their daily life: how they experienced being responsible for their younger siblings, how it felt to be different from many other children (with parents) in their village, and how they experienced being part of their village community or excluded from it. In this study, quantitative analysis was less significant than understanding the children's experiences of their daily lives and the means to contribute to the children's greater inclusion in the life of their village. We felt that the use of qualitative in-depth interviews, focus group discussions, narratives and observation methods would best achieve these understandings. The interviews and the focus group discussions were carried out mainly in the local language, Luganda. Some heads of households wrote autobiographical narratives.

Villages in which we conducted data collection were selected randomly. We then visited the chairmen who gave us permission to conduct the data collection in their villages of responsibility. Most chairmen had substantial knowledge of the inhabitants who lived in their village, and thus they assisted us in finding the sibling-headed households. All participants were selected using convenience and snowball sampling, i.e. the chairmen pointed out some, others were referred to by those interviewed. The children who wrote narratives were literate and were picked randomly among heads of households we contacted.

The data collection was conducted with 43 heads of households. Twenty-one were interviewed individually, 16 participated in focus group discussions and six heads of households wrote narratives only. The focus groups were gender-mixed for most discussions but for discussions of intimate topics, we divided the groups into males and females. These groups were respectively facilitated by a male and a female research assistant. In total, eleven orphans wrote narratives, five of whom were also interviewed or participated in focus group discussions. In order to write these narratives, the orphans were given a notebook and a pen and asked to write about their daily lives, how people treated them, their major concerns, their view of their future, and of what could improve their lives. After five to seven days, we returned and collected the notebooks. The narratives were mainly written in Luganda and translated into English. We also included in our findings information derived from informal discussions with chairmen.

The interview- and focus group discussion guides included questions about socio-demographic variables such as the presence of food and shelter, sources of income, educational level, school attendance and school performance. Questions about close friends and relationships to other people and the experiences of stigma were investigated in depth. We also emphasized issues such as reasons for and ways of dealing with sadness, experiences of and reasons for happiness, coping strategies, dealing with siblings and general difficulties and challenges. A flexible design allowed us to add or modify questions in order to clarify possible divergences. Tape recording was not feasible, but the primary researcher and two research assistants who took part in interviews and focus group discussions wrote copious notes. During the interviews, the primary researcher kept a comfortable distance away from the interviewee and interviewer to avoid discomforting the child.

Analysis during data collection and a thorough review after each interview/focus group discussion gave us the opportunity to "cycle back and forth between thinking of the existing data and generating strategies for collecting new data", see page 50 [30]. Some of the informants offered responses which created new issues and which we included in subsequent interviews and discussions.

The analysis followed four steps: 1) reading the material to obtain an overall impression; 2) identifying units of meaning representing different aspects of interest, and coding these aspects; 3) condensing and abstracting the meaning within each of the coded groups; and 4) summarizing the contents of each code group to generalize descriptions and concepts reflecting perceived important experiences of interest [1,31].

The subsections in the findings below were decided upon during and after the data analysis. Our aim was to emphasize the children's psychosocial challenges. However, we realized that the explanations for such challenges were extremely complex. Thus the children's' various daily problems are presented first and then are followed by descriptions of how different challenges impact the heads of households' psychosocial conditions.

## Results

A multi-method approach, with each method having unique strengths, allowed a comprehensive understanding of the orphans' situation. Information about the heads of households who were interviewed (21) is more detailed than that about those who participated in focus group discussions (16) or those who were narrative writers only (6), but the combined sets of information gave a fuller perception of the lives of these orphans than would have been offered through the use of any one of these methods alone.

### Age and household composition

The heads of households participating in the study were between 10 and 21 years old, with the average age of 15.6 years. The six heads of households who participated by only writing narratives gave no information about the number of siblings they took care of in their homes. The total number of children in the other households (37) was 123 and the mean number of children in each of these households was 3.3, which is higher than the official 2.8 mean number of children in child-headed households [15]. This may be explained by the fact that we included heads of households older than 18 years old. The younger, non-head of household, siblings' ages were between 1 and 16 with a mean age of 11 years. One of the households took care of an unrelated physically disabled orphaned neighbor boy. Two boys each lived alone with their disabled grandmothers and one girl lived with her siblings and their old and sickly grandfather. Eight children each lived alone.

The children stayed in their parents' homes for different reasons. Some said they were afraid that relatives or other people would take their properties if they left their homes. Others explained that there was no other place to go. A few had tried to get some help and wanted to live with relatives but they were refused; others said they had promised their parents to stay in the house to take care of their belongings. The siblings tried to avoid being split up by being taken care of by different relatives. A few orphans were migrants from Rwanda and could not trace their relatives. Their parents had been refugees due to the 1994 genocide.

Twenty-five male and 18 female heads of households participated in this study. According to responses from the chairmen who were contacted, there were more boys than girls who headed households. The reason for this skewed distribution was that some of the girls had married young - or had just left the community: *"Our eldest sister lives in Kampala. She left home without telling anyone where she was going. Ever since 2002 she has never come back" *(Boy, 16). Most of the children whose older sisters or brothers had left the home did not know why or where they had gone. The chairmen verified that more adolescent girls than boys left their homes and explained that for some, especially girls, the burden became too heavy and an easy way out was to work for other people or just "go to town" (Kampala, the capitol city).

### Cause of parental death

We asked the 21 interviewed children this question to find out what they themselves believed to be the reason for their parents' death. We realize that the children's belief and the actual cause may be different. Sixteen of the 42 parents were reported by the children to have died due to HIV/AIDS. However, the number could be higher as most of the parents were said to have died of unknown or vaguely described causes such as fever, headache and witchcraft.

### Shelter

During our visits, we observed the conditions of the sibling-headed households. Ten of the heads of households said that their houses needed to be repaired or they reported that they *"want [ed] a house constructed" *because they lived in a shattered house or temporarily with friends due to lack of housing. We observed that at least one third of the participating children's houses were in very bad and dangerous conditions; some had already fallen down or had collapsing walls and leaking roofs i.e. much worse than the average quality of houses we observed in the district.

There were great differences among the households visited, ranging from possession of one bed, one mattress and one blanket for each child, to four or more children having to share one bed. In most households, the children had nothing but one bed sheet or one blanket to share. Many used their own clothes as a mattress. According to the children no one had mosquito nets, nor did we observe any.

### Food and maintenance

In three of the sibling-headed households the children had had no food in the last 24 hours. In seven households the children usually had two meals or more per day. None of them could afford to eat fish or meat.

All the heads of households reported that they struggled to obtain food, water, paraffin and firewood. For most of them, the lack of these basic needs caused worries. Some of them were very young when their parents had died and therefore they were not skilled in taking care of their house and garden. They tried to cultivate and grow crops as well as they could, but even that created challenges for them. As one of the children wrote in her narrative, *" [cultivating] coffee helps us in satisfying our needs but the bad people come in the plantation and get off all the ripe ones when we are at school and even the little we stored in the house was stolen as they broke the window and took a barrel of dry coffee"*. Domestic animals were also reported stolen. Further, if the children were not at home during the day to look after their gardens, crops were taken by monkeys or neighbor's animals.

### Workload

The children in the sibling-headed households had to work for other people to earn money. This included garden work, looking after animals, fetching water and firewood, and washing cars and motor cycles.

The heads of households had a tendency to compare themselves unfavorably to other children, for example, when they saw other children who had parents able to give them the necessities of life, the orphans felt different and less valuable. In addition, the fact that the children in the sibling-headed households had to work for other people to survive gave them a feeling of having a very low status and self-esteem.

### School attendance

Fifteen of 37 heads of households (six narrative writers not included here) had stopped school, most of them after primary school. They cited lack of school fees as a great challenge. However, they were much focused on education and felt that skills and knowledge obtained in school could help them achieve a better life. All the heads of households participating in this study reported that they needed help to pay school fees and buy scholastic materials. World Vision (WV) paid half the school fees for six of the interviewed heads of households so they could attend secondary school, yet some of them still didn't attend school because they were not able to raise the other half of the fees. Some also argued that WV had stopped sponsoring them in the middle of Secondary school. A few teachers and community members tried to help some of the brightest orphans, while others assisted the neediest ones.

Children were often sent home from school for not paying fees, not having a uniform or for other reasons; *"I am always chased from school because of no school fees. I blame this on the death of my parents"(Boy, 14)*.

Among the 21 heads of households who were interviewed, five attended the class grade corresponding (+/- one year) to their age. Others were in a class grade two or more years below their age. The study did not determine whether this was considerably different from what is usual for all children in Rakai District.

The heads of households themselves argued that if they had to do some work in the morning that would cause them to be late for school; they would rather stay at home the whole day because they were punished and spanked if they came to school late. One boy said, *"I wish they [the teachers] would listen to us and try to understand our problems instead of just spanking us." *Some heads of households argued that if they attended school but could not pay the fees, they would not be given their school reports, which again prevented them from continuing to the next grade.

Four heads of households held a responsible position at school such as prefect or class monitor or to *"keep the key for the staff room"*. All these had sponsors who paid for their school fees. Reasons for others not being voted into such positions included lack of school fees, irregular attendance, no uniform, etc. One 14 year old boy stated that: *"I couldn't pay my class mates to vote for me"*.

### Health

The children in the sibling-headed households said that they were not more often sick now than when their parents were alive. In principle, their access to health facilities is the same as that of other children, but lack of money for transport and medication often hinder them from receiving medical care. Relatives and some community members, but mainly the chairmen, supported some of the children when they were sick. The children's chances to get assistance from other people when sick differed from one place to another.

### Children's worries and sources of happiness

The main concerns of the heads of households were worries and anxieties about basic needs. To determine the predominant psychological factors affecting these children, questions were asked about whether or not they slept and ate well (appetite) and if they could describe what caused their different moods.

Due to knowing their parents died from HIV/AIDS, many heads of households were afraid: "*I begin to think I am going to die too, and I find myself very worried and not fit to keep company with my friends" (Boy, 16)*.

The heads of households reported that they and their siblings felt happiness when they could attend school, when other people treated them well and when teachers did not beat them. Many of them claimed that *"I am happy when we have some food and when my siblings or Jjajja (i.e. grandmother) are not sick" (Boy, 13)*, or *"when we are healthy and alive" (Girl, 11)*.

The question about whether the heads of households and their siblings missed their parents was not directly asked. On the surface, it seemed as if they did not think about their parents a great deal but the effect of the loss became apparent in situations when they faced a problem that they thought their parents would have easily solved. Most of the children said that they became sad when they were thinking about their parents; *"I find myself crying, especially when we are eating. I remember my mother and cry. My sister cries too, especially when I am sick. She thinks I am going to die like mama did" (Boy, 12)*.

Most of the heads of households pointed out that the situation had become worse after the death of their parents: *"All the time we live in uncertainty about the future. We left our homestead, the house collapsed, it was very old. Ever since our parents died, we are suffering" (Boy, 16)*. Some of them were overwhelmed with additional challenges after their parents had passed away as this boy explains; *"No adult looks after me. All my relatives died. It is me now, looking after my grandmother" (Boy, 13.) *A few were facing so many difficulties that they were thinking of leaving home:"*I felt something within myself that told me I should leave and look for a different place to stay, but I have nowhere to go...and many times I would say that God was very, very unfair for I did not know what plans He had for me in this world....He seemed like He was not hearing" (Girl, 16)*.

The quotations given above are but a few examples of many similar expressions. However, a few orphans said that they were getting used to the situation and felt confident that they could manage. One 16-year-old boy claimed that he had no problems and that "*life is good"*.

### Friendship

Most of the heads of households and their siblings tried to continue being active in sports and other social activities. But while these interests remained intact, greater responsibilities at home reduced the possibilities to "nurse" friendships; *"I used to play with friends but ever since I lost my parents I spend most of the time working to get money" (Boy, 14)*.

Orphans who managed to go to school seemed to have more friends than others; "*I have friends at school but not at home" (Boy, 12)*.

### Social interaction and its implications

During both the data collection and times we were assisted by chairmen, we observed that in most villages, the chairmen were aware of the orphans' situations and gave them much attention and assistance. However, in a few villages, the chairmen were quite ignorant about the situation in the sibling-headed households and in one specific village, the chairman was rather negative about these orphans. When we gave a hint that HIV/AIDS might have caused the death of many people, this chairman responded that *"people have always died from pneumonia and tuberculosis and they still do. There is no difference. There is no AIDS"*. From this and other comments, we realized that lack of knowledge about HIV/AIDS includes even denying the existence and implications of the epidemic.

The quality and frequency of social interaction with other children, teachers and community members contributed to the heads of households' experience of the extent to which they felt included in the community. The fact that they had very limited contact with their relatives seemed to be the most painful and least understandable; the "why me?" question was often asked. The children also said that adults did not seem to understand the problems the children face every day. In many situations the heads of households were not able to understand why people ignored them or did not try to help them. A 15 year old girl told us that *"some people can help by giving me some leftover bananas, but there is someone who can have enough food but gives it to a pig"*. The few heads of households who received help from relatives or other adults emphasized their gratitude: *"it shows that somebody cares" *and *"it shows me how much they like me and care for me" (Girl, 11)*.

Most of the children lived without any adult care but some said that community members or relatives would at times give them some food or money. Among the 21 heads of households interviewed, twelve reported that no adult ever came to look after them or assist them. A few children had tried to visit relatives hoping to receive some help, but were refused. Only one head of household said that she and her siblings were looked after and cared for every day, and one boy claimed that their grandfather came and stayed with them for a week now and then. Among the other seven sibling-headed households, four received weekly visits, two within the last four months and one had not been visited since more than five months ago. One boy (15), taking care of two siblings, said that "*We would wish that friends and relatives could visit us. Our relatives never visited us since our mother died and yet we visit them. We need someone in the village to comfort us and we hope that we can continue to go to school"*.

The ones who offered help were often relatives, but most children referred to the chairman as an important person in their daily lives; *"Chairman gives me medicine when I am sick" *and *"When we need some help in the house, we can ask chairman and he will assist us" (Girl, 17)*. However, there were huge differences between the various villages when it came to whom the children mentioned as the main helper. One head of household said that; *"Some people don't even greet us...many people, they don't care what happens to us" (Boy, 16, in focus group)*. Two heads of households reported that they were being helped by Catholic nuns.

In one case, the head of household (girl, 17) argued that the children in the orphan headed households had difficulties because their relatives tried to take their properties; "...*but we deal well with our relatives after a clan meeting initiated by the chairman"*.

With a few exceptions, the heads of households said that they related well with their siblings; *"We discuss about what each of us has to do." *Only a few reported that they played with other children and that they had friends. Most had friends who were in similar situations; *"My friends treat me well; this is because most of them are also orphans"*. However, some experienced a different attitude from other children who did not quite understand why the heads of households and their siblings had to work as hard as they did. *"When they see us digging for other people they ask why, so we tell them that our parents died."*

In other cases, the orphans in sibling-headed households wished that other children and people did not know they were orphans because it caused difficulties. Some said that many people pointed fingers at them saying, *"her parents died of AIDS; she too, the child has it!" *Others preferred not to talk to their friends about their situation: *"We don't talk to them, because they can't do anything about our problems. But we play with them" (Boy, 12)*. We found that only one head of household had a confidante (maternal aunt) with whom to discuss difficult personal issues. Some mentioned that they could talk with the chairmen or a neighbor, and one girl (15) said that *"we consult any adult who gives us advice and comforts us... [but] we don't discuss personal things"*. Other heads of households said that they would rather not talk to other people about their situation. Some of them did not regard other people or relatives as potential confidantes: *"We relate well with the community members but we do not communicate about our problems" *(Boy, 12).

At times the orphans in the sibling-headed households seemed to misunderstand people's well-intended approach. One chairman said: *"When we try to guide the orphans and tell them to go to school, they believe that we yell at them just because they are orphans. They do not understand that we try to help and guide them" *and another said: *"They refuse to hear advice given to them when they do something wrong. At times they run away from school even when they are advised to stay"*.

### Coping

In order to find out how they coped in their daily lives, we asked the children what they did when they felt sad or angry and how they handled situations when they felt lonesome or worried about the future. Though they reported various approaches to coping often the heads of households coped with worries and sadness by praying, talking to siblings or other people, or just going to sleep or doing work. Mostly, they kept to themselves.

When we asked the heads of households about their psychosocial *needs *they talked about what they experienced as their psychosocial *problems*. The feedbacks from the heads of households are therefore mainly presented as the practical *problems *and *challenges *they faced. For example, in order to meet basic needs and school necessities, they either worked in their own gardens or worked for other people. When it came to behavior, the children themselves argued that their behavior was good. This is in accordance with other studies which have looked into orphans' behavior [32]. However, according to most chairmen, some orphans in sibling-headed households stole and others, especially girls, supported their families by having sex with men. Some chairmen claimed that the boys were naughtier and that girls seemed to be more polite as their bad behavior was not so noticeable. According to the chairmen, people's opinions about the behavior of children in sibling-headed households varied from one village to another. This had more to do with the characteristics of the communities than of the orphans.

However, in response to the question about psychosocial needs, some heads of households in the focus group discussions did offer suggestions about possible and wished for interventions. One of the heads of households said that: *"we need help to bring understanding between the community members and us, so that we can live happily and not hate each other" (Boy, 17)*.

## Discussion

The sibling-headed households in this study were composed of children living in exceptionally difficult circumstances. The socio-demographic conditions such as gender, school attendance, sources of income and basic needs, were relatively identical for all the sibling-headed households, though the quality and quantity of household goods differed. The heads of households' psychological situations depended on a number of factors, including the degree to which they received practical and emotional support from relatives or members of their village, the area in which they lived, the number of siblings per household, whom the heads of households had to care for and the possibility to attend school.

Both socio-demographic and social conditions had a deep impact on these orphans' psychological wellbeing. Bereavement and grief are also relative to context and the child's developmental stage. For example, Bowlby's four phases in a grieving process--shock, protest, denial and recovery [33]-- may turn out very differently in different contexts and in accordance with the person's possibility of having social interactions within family, school and community [34]. In understanding needs and challenges, quite a few studies emphasize the importance of being aware of contextual differences, such as stigma, faced by children orphaned (or affected) by HIV/AIDS. However, there are *"commonalities in what causes stigma, the forms in which stigma is expressed, and the consequences of stigma"*, see page 37 [35].

The children in the sibling-headed households, and especially the breadwinners (heads of households) with their grown-up responsibilities, required adult guidance regarding social and psychological issues as well as practical skills in such tasks as cultivating gardens and maintaining their houses. Those who received attention and/or assistance from relatives or other adults showed gratitude and seemed to feel proud, and they functioned better than the others. It gave them the sense of having someone to belong to and someone who cared, a feeling they indicated to be very important because their parents were dead and because they compared themselves with other children who lived in intact families.

The information gathered made it clear that the chairmen, with some grave exceptions, showed a greater sense of responsibility and understanding than most of the relatives and other community members. This might be explained by the chairmen's relatively frequent contact with people from outside the village and the district, and their access to newspapers and official documents which made them more aware of the problem of HIV/AIDS and its impacts on individuals and communities.

The villagers' sense of responsibility and their understanding of the challenges that the orphans faced varied hugely from village to village. The quality and degree of assistance provided by the chairmen and other village members in caring for the orphans and the relationship to the sibling-headed households correlated well with the knowledge about both HIV/AIDS and the situations in the sibling-headed households. The chairmen with less interest in the sibling-headed households seemed to have "convinced" the village members to have the same opinion towards the sibling-headed households as themselves. In one specific village, the chairman called these orphans the "COTOs" - or the "Children On Their Own." In that village, the children's worries were greatest and their situations worse than elsewhere when it came to interaction with other people. The chairman's attitude might have contributed to additional marginalization of the orphans in these sibling-headed households and also increased power inequity [36] between the orphans and the rest of the community.

Lack of information and skills, seen in the behavior of the chairman who did not know about HIV/AIDS, might explain the different treatment of the children by members of their village. This behavior might be either a remarkable display of ignorance of the facts regarding an epidemic which has been in existence in the area for more than 25 years, or it may be seen as an expression of total denial. Another explanation might be the experience of powerlessness felt by the village leader who lacked sufficient means to assist the increasing number of orphans, in addition to facing other challenges and problems in the villages. No official government initiatives or agents helped these orphans.

Since 1997, the Universal Primary Education (UPE) program in Uganda has provided tuition free primary schooling for all children but only to Primary Class 7, which explains why some children stopped attending school after that level. And even in the classes where tuition is free, the pupils still have to pay for other costs, such as scholastic material, uniforms and the like. Tuition in some Vocational Institutions is free but this kind of education requires that the students pay for scholastic materials. Many of the vocational institutions are residential, which require payment for room and board. The UPE program does not give concessions for children with special needs such as orphans or those who have to take care of sick parents or relatives and siblings [10]. According to Luzze and Ssedyabule [10] 12.5% of children in child-headed households in Rakai are not going to school and 61.5% of heads of households are not in school. According to UNICEF the percentage of children attending school at the national level in Uganda is 81.5% but only 48% of all children reach grade five [37].

The negative consequences of not attending school deeply affect the orphaned children. In addition to having lost their parents and thereby the possibilities to a "normal" childhood, they had little or no social interaction with other people that schools provide. The teachers at the schools were in a position to give the children some of what they could not acquire from parents. As some of the heads of households claimed that they had friends at school but not at home, they lacked another important social "link" when they were deprived of the possibility to attend school. All the heads of households were convinced that education is important. They seemed to have understood that interrupted schooling probably would mean increased poverty, and the oldest orphans also saw how this could increase the risk of HIV infection [38,39].

Significantly, despite the ever-present challenge of obtaining sufficient food, and their more or less constant feeling of being hungry, many heads of households said that their appetite was not good and they slept poorly. Both sleeping difficulties and loss of appetite were among the common symptoms of distress observed in these children. Research suggests that child hunger itself can have significant negative physical and mental consequences [40]. However, severe hunger was not found among the orphans in this study.

A recent article based on a study by Evans and Schamberg (2009) "...show [s] that childhood poverty is inversely related to working memory in young adults. Furthermore, this prospective relationship is mediated by elevated chronic stress during childhood", see page 6545 [41]. The double orphans living in sibling-headed households are not only poor, but appear to be living with a considerably higher level of stress than other orphans, and certainly more than other children living in intact families, whether in poverty or not. Consequently, they seem to be particularly vulnerable to suffer from having working memories that are negatively affected and they therefore might find it more difficult to learn than other children. Such biomedical research is another verification supporting the assertion that double orphans living in sibling headed households are particularly vulnerable.

Many countries affected by the HIV/AIDS pandemic have experienced changed social structures. For example, in regions affected by the pandemic, the supportive ability of the extended family has to a great extent collapsed although people are still culturally and mentally attached to a collectivistic communal way of living [42,43]. A social structure such as eating or sleeping alone was not common before the pandemic and probably presented serious problems to the double orphaned children in Uganda, giving them a feeling of extreme loneliness.

Negative experiences in their earlier lives, such as the death of parents, as well as the heads of households' current precarious living situations, led to constant preoccupation with how to survive. These conditions contributed significantly to psychological distress. There was thus an interrelationship between the socio-demographic- and social circumstances and the heads of households' psychological conditions which caused substantial anxiety among them and their siblings.

The lack of caring adults or "significant others" led to incomplete socialization for the children in the sibling-headed households. The fact that these orphans mostly were considered HIV/AIDS victims also compromised their social interactions.

Obtaining psychological, social and cultural competence [44] required for these children's new way of life had been seriously limited. In "normal" settings, children can manage to successfully survive the different phases of their grieving process [33] because they are cared for and protected by other relatives or friends. But the interactions, situations and social systems that usually support children's developmental course [34] are far less available to the orphans in our study, and absence of resources leads to disrupted or impeded grieving and socialization processes. Children in poor areas who are orphaned due to HIV/AIDS may face a chain of negative events and difficulties in addition to the loss of both their parents because, in addition to stigmatization, they must also sacrifice education, friends and other socializing activities.

The importance for the children to become socialized to and included in their society cannot be emphasized enough. These children struggled to be the same as other children but were in many cases treated as undesirables. The consequences of not being wanted and of not having parents or friends to guide them hinder or prevent the natural development of these children and their future children. As Alisdair MacIntyre [45] has claimed:

*We become independent practical reasoners through participation in a set of relationships to certain particular others who are able to give us what we need. When we have become independent practical reasoners, we often will, although not perhaps always, also have acquired what we need, if we are to be able to give to those others who are now in need of what formerly we needed. We find ourselves placed at some particular point within a network of relationships of giving and receiving in which, generally and characteristically, what and how far we are able to give depends in part on what and how far we received*. See page 99 [45].

Link and Phelan [46] suggest that the "multifaceted and dynamic processes" brought about by social conditions as well as by lack of resources such as money, knowledge, power, prestige and interpersonal resources, often have a strong connection with disease. This connection has also recently been emphasized by the final report of the UN Commission on the Social Determinants of Health, chaired by Michael Marmot [13]. For orphaned heads of households, these negative social conditions and lack of resources put them in a particular "risk of risks" situation. Their risk of getting diseases, including HIV/AIDS, can increase, even if they have knowledge about prevention, because they are poor and deficient in positive social interactions. HIV/AIDS is a threat, especially to the girls who might be forced into survival sex because of lack of options and means of livelihood for their siblings and themselves [16,47,48]. Also, as more adolescent girls than boys leave their homes because they are overwhelmed by the present situation and as it is easier for girls than for boys to establish themselves elsewhere, more girls than boys can end up in situations which put them at greater risk of getting HIV/AIDS [49]. "HIV is also a major risk for children who already lack parental care, whatever the reason. Children living on the street or in other unprotected situations are likely to be at greater risk of HIV due to factors such as low self-esteem, poor socialization skills, and lack of vocational skills, which increase the likelihood of risky behaviors as well as of abuse", see page 2 [18]. According to The Uganda AIDS Commission [50] "many street children are orphans who left their homes in search of company and new ways of survival", see page 11 [50].

Our research tools were not suitable for measuring the exact levels of self-esteem, but our observations led us to conclude that the heads of households' self-esteem was very low; most of them explained that they had problems coping with the challenges they faced and showed little happiness other than when they were given material assistance. Thus, their self-esteem could be high for a short period but was fragile, materially conditioned and not sustained. Their view about the future as hopeless, or, uncertain and worrying, was not in concordance with having a high self-esteem [51].

The heads of households' coping strategies varied. According to MacIntyre [45], coping strategies depend on people's nature, the extent to which they have learned from others while growing up, and also how they have been treated by society. Some of the heads of households were much stronger than others, with higher self-esteem and greater initiative. These orphans lived in villages where the chairmen and the community members were more supportive than in other places. The quality and quantity of social interaction, and having social relationships [52,53] was critical for their future physical, social and emotional health [54].

An interesting research finding is the contradictory views about the heads of households' behavior. These children themselves argued that their conduct and behavior was good but most chairmen were of a different opinion. However, it is not difficult to understand that children may report themselves as having good behavior even if they actually don't. And it is important to note that "bad behavior," such as stealing, is often a result of hunger and lack of outside assistance.

## Conclusion

In this article we have presented practical, material and psychosocial challenges faced by double orphans heading households in randomly selected villages in Rakai District in Uganda.

The main aim has been to illuminate these children's psychosocial challenges. To a great extent, these challenges are the consequences of the many, and often multifaceted problems they face, leading them to experience "survival anxieties". Another significant cause of these challenges is their village members' lack of information, understanding, or means and capabilities to support the children. This circumstance leads to stigmatization of the orphans: they are "cut dead" and "cut off".

Studies on children affected by HIV/AIDS started relatively late compared to studies focusing on other aspects of the pandemic. Most studies with focus on orphans' psychosocial challenges have explored the situation among orphans in school, in care arrangements, and among those supported by various NGOs [9,10,24,32,55]. Much has been learned from research carried out to date, but more needs to be learned because it is vitally important to effectively address these children's needs. [56]. In addition, stigmatization and discrimination must be combated in order to reduce HIV and AIDS and consequently, the overall number of orphans [36,56,57].

It is a complex challenge to try to help the children in these difficult circumstances towards a better future. All the members of their villages should be involved in this effort, but this inclusion requires that villagers understand, accept and respect the children in sibling-headed households and their situation. In their study carried out in Rwanda, Thurman et. al. [55] describe the difficulties of community involvement and the need to carefully and thoughtfully plan for this before implementation.

In addition to recommending contributions from community adults and teachers, the information presented in this paper will provide baseline information to carefully discuss, plan and initiate interventions to improve the situation for the orphans and their communities and to reduce stigma in Rakai District in a sustainable way. Another major recommendation is that sponsors and local government be encouraged to initiate action to meet the orphans' request for education. In our study, the orphans emphasized education as one of the most important means for improvement. Education would give them the possibility to earn money and to strengthen their socialization into an adult world. At the same time, it is also important to find other "alternative opportunities ... for the older orphans rather than forcing them to join formal schools", see page 49 [58]. Small loans or help to start income generating projects would be a reasonable option for this group of older orphans.

Sending the older siblings to school requires that other people take care of the younger ones in the sibling-headed households. This is another reason why it is very important that community members become much more involved. In order to influence and increase community members' degree of participation and concern for the orphans, we believe that thorough information and counseling is necessary. According to Thurman et. al. [55] "The way forward for children in difficult circumstances is not just by the direct provision of services. The rhetoric of community-based care must be translated into real interventions that foster the nurturing environment that children need. Energy and resources must be allocated towards creative and context-specific approaches that engender community support", see pages 1556-57 [55].

After nearly 30 years of the pandemic, to a tremendous extent we still see stigmatization and lack of knowledge. Without intervention the situation of orphans will remain difficult and will too often end disastrously, especially in low income countries of Sub-Saharan Africa such as Uganda. Even if the HIV prevalence in Uganda (or elsewhere) were decreasing the actual number of people living with HIV/AIDS might increase due to population increase. And, even if HIV testing and medication become free, the number of parents dying might increase due to lack of knowledge and due to stigma related to the disease (i.e. they do not know about it or they do not dare to go for testing and medication).

We will use the data gathered by this study to initiate interventions and to encourage others, including governments, to do the same.

It is, of course, the local and national governments which should take the lead to improve the situation for orphans and other vulnerable children. It is their responsibility to provide education and food, and to pass and enforce laws to protect the children against stigma, abuse, property grabbing, and other forms of mistreatment. We hope that this paper will contribute to illuminating the needs of orphans in sibling-headed households and to actions that will help relieve their suffering.

## Competing interests

The authors declare that they have no competing interests.

## Authors' contributions

ND designed the study, organized and led the data collection, wrote field notes, analyzed and interpreted the information collected and drafted the paper. SM assisted in the preparations of interview and focus group guides, supervised the data analysis and critically reviewed the paper. AJN carried out most of the interviews and focus group discussions, translated interview and focus group guides from English to Luganda, prepared data and translated all information from Luganda to English, and reviewed and commented on the paper. All authors have read and approved the final manuscript.

## Pre-publication history

The pre-publication history for this paper can be accessed here:


